# Automatic Detection and Classification of Diabetic Retinopathy Using the Improved Pooling Function in the Convolution Neural Network

**DOI:** 10.3390/diagnostics13152606

**Published:** 2023-08-05

**Authors:** Usharani Bhimavarapu, Nalini Chintalapudi, Gopi Battineni

**Affiliations:** 1 Department of Computer Science and Engineering, Koneru Lakshmaiah Education Foundation, Vaddeswaram 522302, India; 2 Clinical Research Centre, School of Medicinal and Health Products Sciences, University of Camerino, 62032 Camerino, Italy; 3 The Research Centre of the ECE Department, V. R. Siddhartha Engineering College, Vijayawada 520007, India

**Keywords:** CNN, diabetic retinopathy, fundus image, pooling function

## Abstract

Diabetic retinopathy (DR) is an eye disease associated with diabetes that can lead to blindness. Early diagnosis is critical to ensure that patients with diabetes are not affected by blindness. Deep learning plays an important role in diagnosing diabetes, reducing the human effort to diagnose and classify diabetic and non-diabetic patients. The main objective of this study was to provide an improved convolution neural network (CNN) model for automatic DR diagnosis from fundus images. The pooling function increases the receptive field of convolution kernels over layers. It reduces computational complexity and memory requirements because it reduces the resolution of feature maps while preserving the essential characteristics required for subsequent layer processing. In this study, an improved pooling function combined with an activation function in the ResNet-50 model was applied to the retina images in autonomous lesion detection with reduced loss and processing time. The improved ResNet-50 model was trained and tested over the two datasets (i.e., APTOS and Kaggle). The proposed model achieved an accuracy of 98.32% for APTOS and 98.71% for Kaggle datasets. It is proven that the proposed model has produced greater accuracy when compared to their state-of-the-art work in diagnosing DR with retinal fundus images.

## 1. Introduction

Glucose in the body is converted into energy, which helps with everyday tasks. Diabetes is caused by obesity, poor nutrition, and limited physical activity. However, elevated blood glucose can build up in the blood vessels of several human organs, including the eye. People who have had diabetes for over a decade have the chance of getting diabetic retinopathy (DR) [1]. Globally, the population suffering from diabetes is expected to reach 552 million by 2030 [2]. Preventing visual loss is possible with early detection and sufficient treatment [3]. DR consists of five classes—no DR, mild, moderate, severe, and proliferative.

DR can affect blood vessels, in severe cases damaging, enlarging, or blocking them, or causing leaks; the abnormal growth of blood vessels can cause total blindness. Micro-aneurysms, haemorrhages, and exudates are the major signs of retinal DR. The level of the disease can be identified based on the shape, size, and overall appearance of the lesions. The main benefits of DR screening are its high effectiveness, low cost and minimal reliance on clinicians (i.e., ophthalmologists). The global eye screening tool for DR is the fundus photograph [4]. To prevent diabetes-related blindness, automated screening allows for clinically convenient and cost-effective detection [5].

From the field of computer science, deep learning can be a practical approach to automatic DR detection [6]. A deep learning system automatically identifies the DR with an accuracy that is equal to or better than that of ophthalmologists. The core deep learning model for medical image diagnosis prediction, and classification is the convolution neural network (CNN). However, there is the possibility to improve the performance of the model by tuning the hyperparameters in these deep learning-based models.

CNN models AlexNet and VGGNet-16 have been implemented for this purpose and the results suggest that VGG-19 performs best; however, the DR stages have not been explicitly ranked [7]. A hybrid technique incorporating image processing and deep learning was proposed for the detection and classification of DR in the publicly available dataset MESSIDOR, and Histogram Equalization (HE) and Contrast Limited Adaptive Histogram Equalization (CLAHE) were implemented to improve the contrast of the image [8]. Other CNN models, like Inception V3, Dense 121, Xception, Dense 169, and ResNet 50, have been explored for the enhanced classification of different DR phases [9].

In another study, the authors proposed a framework with a new loss function by implementing mid-level representations to improve DR detection performance [10]. Another report proved that VGGNet produced higher accuracy compared with other CNN models such as AlexNet, GoogleNet, and ResNet for DR classification [11]. A CNN model implementation with data augmentation for DR image classification was presented in [12].

Other frameworks for the early diagnosis and classification of DR were presented for Grampian [13], MESSIDOR [14], and EYEPACS datasets [15]. In [16], the authors mentioned that 90% of accuracy was achieved in diagnosing microaneurysms and extracting and classifying the candidate lesions. All of these existing studies have implemented built-in hyperparameters. However, model performance can be improved by adjusting hyperparameters within deep learning models. To counter the self-strengthening trend and ensure that as many candidate component models as possible have been properly trained, we have added balance loss to our model. The proposed approach could extract key features from the fundus images that can help make an accurate DR diagnosis.

## 2. Materials and Methods

**Figure 1:**
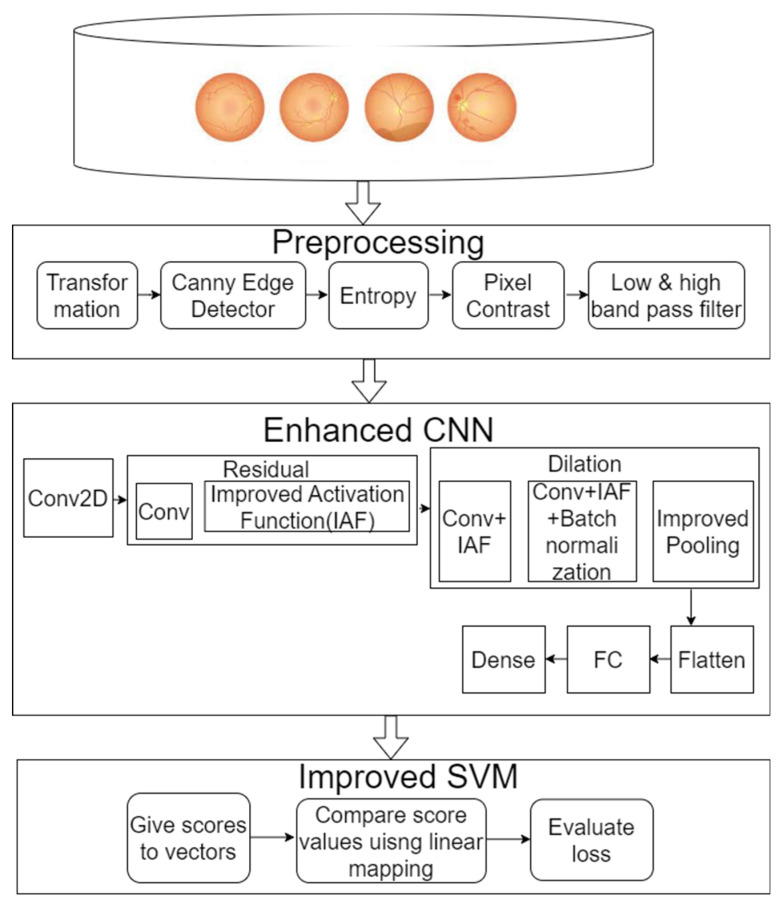
Experimental framework.

The objective of the current study was to accurately categorize DR fundus images into different severities. We discussed an automated system for assessing the seriousness of diabetic retinopathy. The classification accuracy for diabetic retinopathy was improved in the current research using a modified CNN architecture. Figure 1 illustrates the proposed framework.

### 2.1. Dataset Collection

**Figure 2:**
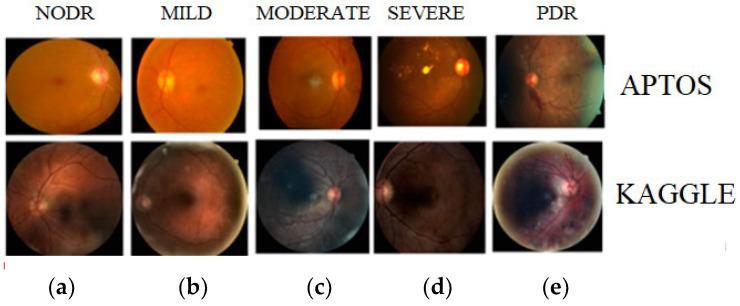
Multiclass of DR (**a**) NODR, (**b**) Mild DR, (**c**) Moderate DR, (**d**) Severe DR, and (**e**) PDR. (First row—APTOS dataset, second row—Kaggle dataset).

We collected the dataset from two publicly available fundus image datasets, i.e., APTOS [17] and Kaggle [18]. Table 1 tabulates the count for five categories in APTOS and Kaggle datasets. Figure 2 shows the sample fundus images from the two datasets. The first-row fundus images are from APTOS and the second-row fundus images are from the Kaggle dataset.

**Figure 3:**
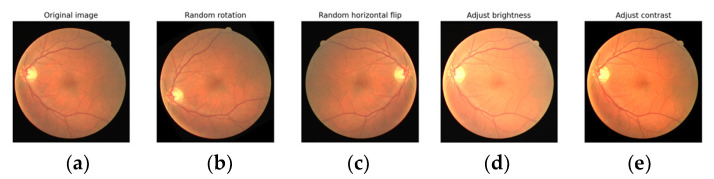
Augmentation (**a**) Original image, (**b**) rotation, (**c**) horizontal flip, (**d**) brightness, (**e**) contrast.

We employed data augmentation to increase the number of images throughout the training sample. Once provided with more DR to learn from, DL approaches generally improve their performance. Overfitting is avoided and the imbalance in the dataset is corrected by the application of data augmentation. Horizontal shift augmentation was one of the transformations considered for this study; it involves horizontally shifting an image’s pixels while maintaining the original image’s perspective. The dimension of this transition is specified by a number ranging from 0 to 1 and the viewing angle of the original image is preserved. The image can also be rotated with an additional type of transformation by a random amount between 0 and 180 degrees. By employing data augmentation methods, we were able to fix the problem of varying sample sizes and convoluted categorizations. After augmentation, the APTOS dataset classes were evenly distributed for the training set—1805 for NODR, 1850 for Mid, 1988 for Moderate, 1737 for Severe, and 1770 for PDR. After augmentation, the Kaggle dataset classes were evenly distributed for the training set—25,810 for NODR, 24,430 for Mid, 26,460 for Moderate, 25,317 for Severe, and 25,488 for PDR. Figure 3 shows some of the augmentation operations followed in this study. Table 2 tabulates the statistics of the data augmentation operations and the final augmented fundus images of each dataset.

### 2.2. Pre-Processing

In this study, we implemented the enhanced artificial bee colony (ABC) algorithm to improve the lesions’ visual contents. Consider $\xi \left(i,j\right)\epsilon D$ with dimensions PXQ, where the values of P, Q are taken as 512 for every image in the database D.

The mathematical representation of the transformation function,

(1) $$\Xi_{f}=\frac{1}{\int_{0}^{1}x^{\left(c-1\right)}{\left(1-x\right)}^{d-1}dx}X\int_{0}^{v}x^{\left(c-1\right)}{\left(1-x\right)}^{d-1}dx,$$

where x is an integration variable and c and d are adjustable parameters of a given function where the maximum value of c is compared with d.

We evaluated the fitness function to adjust the values of c and d and also to measure the complete lesion image.

(2) $$F(\xi_{H}(i,j))=\;\log(\log\left(\sum_{j=1}\left(\Psi \right)\right))M_{\Psi }E\left(\xi_{H}\right)Y\left(\xi_{H}\right),$$

where $\sum_{j=1}\left(\Psi \right)$ represents the total edge intensities of an image evaluated through a canny edge detector. $\;Y\left(\xi_{H}\right)$ represents the contrast of the image $\xi_{H}$(i, j), $M_{\Psi }$ represents the total edge pixels of the processed image, and $\;E\left(\xi_{H}\right)$ represents the image entropy $\xi_{H}$(i, j), represented as:

(3) $$\;E\left(\xi_{H}\right)\;=\sum_{j=0}^{m}q_{i}\log_{2}\left(q_{i}\right),$$

where $q_{i}$ represents the ith pixel intensity probability; the max value is 255.

The contrast of the image is represented as:

(4) $$Y\left(\xi_{H}\right)\;=\;\sum_{j=0}^{mI}Y\left(\xi_{H}\right)\left(I_{i}\right),$$

where $I_{i}$ represents the image blocks and mI represents the mth image block.

The contrasted local band of each block is represented as:

(5) $$\begin{array}{cc} \xi_{Hy}\left(I_{i}\right)\; & =\sum_{\left(p,q\right)\epsilon I}Y\left(\xi_{H}\right)\left(p,q\right)\\ & =\sum_{\left(p,q\right)\in I}\frac{\xi_{H}\left(p,q\right)⊗\phi_{b}}{\xi_{H}\left(p,q\right)⊗\phi_{c}}, \end{array}$$

where p,q represents the pixels of the rows and columns of each block, $\phi_{b}$ represents the bandpass filter, and $\phi_{c}$ represents the low pass filter.

### 2.3. Enhanced ResNet-50

**Figure 4:**
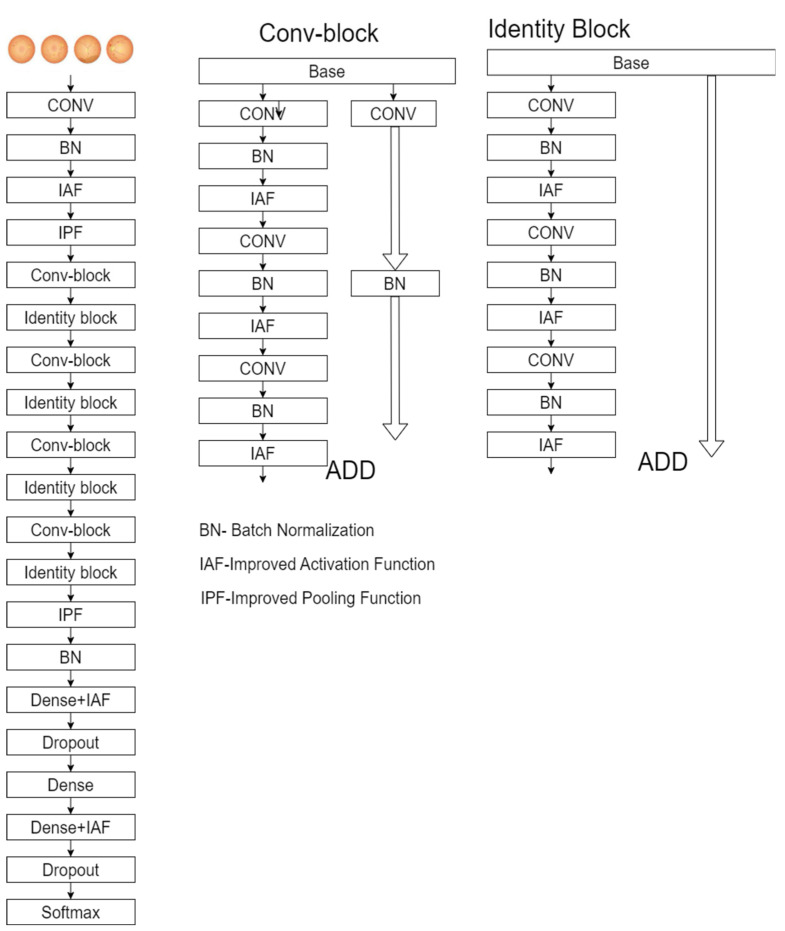
Improved ResNet-50 model.

The proposed model consists of convolution blocks and includes the improved pooling function, a drop-out layer, dense layers, and a SoftMax classification layer; Figure 4 presents the improved ResNet-50 model.

*Convolution Layer:* The convolutional block is the fundamental building component, and each convolution block contains a convolution 2D, an improved activation function, and improved pooling with the average value. The vanishing gradient issue is solved using the improved activation function, simplifying the process so the network can understand and carry out its tasks promptly.

*Kernel:* The model’s initial layer is the convolution layer. This layer initiates the process by applying the filters, also known as the kernel. The kernel size depends on two values—the width and height of the filter. In this study, we set the size of the filter as 3. This filter enables and identifies the features that help understand low-level visual aspects like edges and curves.

*Flattened layer:* The flattened layer is located among the convolution and the dense layers. Tensor datatypes are used as inputs for the convolution layers, whereas dense layers demand a one-dimensional layout. The flattened layer was applied to translate the two-dimensional image representation into a one-dimensional input.

*Dropout Layer:* A dropout value of 0.2 was used in this study, which helps to avoid overfitting. This layer’s function was to turn various components on and off to reduce the model’s complexity and training time. The model thus acquires all the features that are required.

*Dense Layer:* A single matrix is accepted as input by the dense layer, which produces output based on the characteristics of the matrix. The identification and class labelling of fundus images occurs in these layers. The model’s output is produced by a dense layer with five neurons and an improved activation function, and it assigns the image to one of five categories of diabetes: NoDR, Mild, Moderate, Severe, or Proliferative. After a few layers, the proposed activation is applied; this probability-based activation function measures the number of neurons by the entire number of classes.

*Pooling function:* The pooling function in the CNN is primarily used to downsample the feature maps and learn deeper image features that are resilient to subtle local alterations. The features from each spatial region are aggregated in this process. Pooling not only expands the receptive field of convolutional kernels across layers but also reduces memory needs and computational complexity by lowering the resolution of the feature maps while keeping critical features required for processing by the following layers. Pooling can be used in medical image analysis to manage variations in lesion sizes and positions [19,20]. Fundus images frequently have many lesions or parts, which causes their distributions of convolutional activations to be exceedingly complex since unimodal distributions cannot adequately capture statistics of convolutional activations, which limits the CNN performance.

We first pass *Y* throughout a group of prediction layers with parameters $\theta_{p},$ i.e., $c\left(\theta_{p};Y\right)$. The weights are outputted throughout by using a fully connected layer with additional noise.

The improved pooling function is presented as:

(6) $$F_{k}\left(c\left(\theta_{p};Y\right)\right)\;=\;T_{k}^{h}C\left(\theta_{p};Y\right)\;+\sqrt{\delta .log\left(1+exp\left(T_{k}^{m}C\left(\theta_{p};Y\right)\right)\right)},$$

where $T_{k}^{h}$ and $T_{k}^{m}$ are the fully connected layers, the kth parameter and additional noise, δ is the random variable, $C\left(\theta_{p};Y\right)$ are the learned weights, and the weight function can be represented as:

(7) $$w_{k}\left(Y\right)\;=\sqrt{\frac{exp\left(TOP-Q\left(F_{k}\left(c\left(\theta_{p};Y\right)\right)\right)\right)}{\sum_{k=1}^{m}exp\left(TOP-Q\left(F_{k}\left(c\left(\theta_{p};Y\right)\right)\right)\right)}},$$

where *TOP-Q* are the *Q* largest weights.

To make learned weights sparse, we maintained the *TOP-Q* weights and set the remaining ones as negative infinity and we used the improved activation function to normalize all the weights.

We added extra loss using the learned weights:

(8) $$L_{s}\;=3\sqrt{\beta \left(\frac{S\left(\sum_{s=1}^{N}w_{k}\left(Y_{s}\right)\right)}{M\left(\sum_{s=1}^{N}w_{k}\left(Y_{s}\right)\right)}\right)},$$

where $Y_{s}$ is the mini-batch training sample, *S* and *M* are the standard deviation and the mean, and β is the parameter.

The improved activation function, which was recommended as a replacement for the activation function ReLU, is represented as:

(9) $$f\left(x\right)=\;\left\{\begin{array}{c} x/2;if-2\leq x<2\\ -1;\;if\;x<-2\\ 1;\;if\;x>2 \end{array}\right..$$

### 2.4. Classification

We applied the improved SVM in this study to improve classification accuracy. Initially, the SVM calculates the score for all the extracted features by using linear mapping on feature vectors and uses this to evaluate the loss. The improved SVM uses the linear mapping on extracted features to calculate the feature score for the parts of the region of interest used to differentiate the lesion types, which helps in the evaluation of loss function, which helps to obtain the classification results. Algorithm 1 for the improved SVM is presented below.
**Algorithm 1 Improved SVM**    •Initialize the values in the training set.    •Repeat for j = 1 to M. Calculate the loss using the enhanced optimization for all values of j. Compare the extracted regions in the liver images. end     •Repeat for every score vector j − 1 to M. Compute the SVM argmax((w × p j) + b) end 
    •Compute for all weights and finally evaluate the output.

## 3. Results

All experiments were implemented on Keras. The data split was performed based on an 80:20 ratio, where 80% of the data were used for training and 20% for testing. We implemented the proposed pooling function and activation function in the base models VGG-16, DenseNet, ResNet-50, Xception, and AlexNet for the fundus images. Table 3 tabulates the splitting of training and testing sets of fundus images for two augmented datasets.

### 3.1. Image Enhancement Evaluation

**Figure 5:**
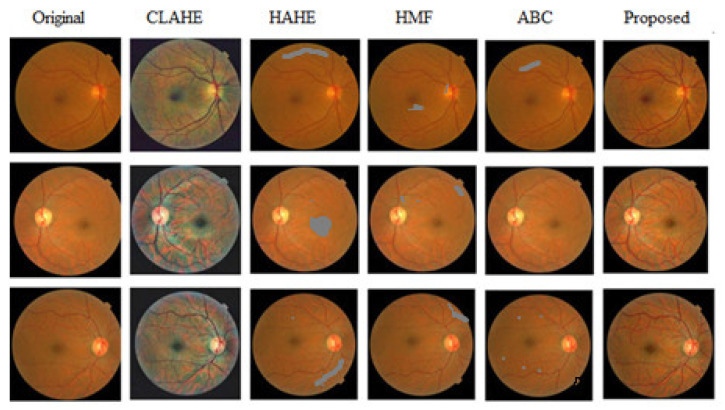
Comparison of the image enhancement of the proposed model with the existing models.

Image enhancement is a vital concept that changes the intensities of the original image to improve the image’s perceptual quality. Figure 5 shows the contrast enhancement results for the APTOS dataset fundus image. Figure 5 compares the proposed model with some other existing enhancement models. Contrast-limited adaptive histogram equalization (CLAHE) models show insufficient image enhancement. The histogram modification framework (HMF) model enhances the image well; however, the hazy look is not adequately removed. The heuristic adaptive histogram equalization (HAHE) model produces an enhanced image with unwanted artefacts visible in the fundus image. The artificial bee colony algorithm (ABC) yields better results than the other existing models; still, it has some viewable artifacts in the fundus image. The proposed model generates an outstanding result compared to all other existing models and successfully improves every minor detail present in the fundus image.

Evaluation and assessment are important for analysing the proposed model performance quantitatively. The proposed image enhancement model is accessed with performance measures such as entropy, peak signal-to-noise ratio (PSNR), the structural similarity index measure (SSIM), gradient magnitude similarity deviation (GMSD), and the patch-based contrast quality index (PCQI) [21,22,23].

Entropy defines the amount of information contained in the processed image.

(10) $$\mathrm{Entropy}=\sum_{y=0}^{255}P\left(n\right)\log_{2}\left(P\left(n\right)\right);$$

where *P*(*n*) represents the probability of the nth level of the image.

PSNR computes the amount of noise content in the processed image.

(11) $$\mathrm{PSNR}=\;20{log}_{10}\frac{2}{\frac{1}{AB},\sum_{x=0}^{A-1}\sum_{y=0}^{B-1}{\left|I_{0}\left(x,y\right)-I_{i}\left(x-y\right)\right|}^{2}},$$

where *A*, *B* denotes the image size.

(12) $$\mathrm{SSIM}=\;\frac{\left(2\mu_{I_{i}}\mu_{I_{o}}+A_{1})(2\sigma_{I_{i}}\sigma_{I_{o}}+A_{2}\right)}{{(\mu }_{I_{i}}^{2}+\mu_{I_{o}}^{2}+A_{1})(\sigma_{I_{i}}^{2}+\sigma_{I_{o}}^{2}+A_{2})},$$

where $\mu_{I_{i}}{,\;\mu }_{I_{o}}$ represents the input and the output intensity values, $\sigma_{I_{i}},\sigma_{I_{o}}$ represent the input and the output standard deviation values, and A_1_, A_2_ represent the constant to limit the instability problem.

Table 4 tabulates the average scores for the augmented APTOS dataset. The performance of the proposed model was demonstrated by comparing six state-of-the-art existing models such as Clahe [24], exposure-based sub-image histogram equalization (ESIHE) [25], HAHE [26], BIMEF [27], HMF [28], and ABC. From Table 4, it is clear that the proposed model achieves a higher SSIM value, and its similarity level is up to the mark when compared with the original fundus image. The proposed enhanced model attains a lesser GMSD value for the images and holds more excellent visual quality compared to the other methods. The proposed model gains a higher PSNR value and the noise suppression level is very good compared with that of the other models. The proposed model holds a higher entropy value to the original image and the amount of information preserved is high compared with the state-of-the-art models. The proposed model obtains a more significant PCQI value compared with the other models, and generates a good quality image with minimum structural distortions. The proposed enhanced model offers less running time when compared to the state-of-the-art contrast enhancement models. The running time of the CLAHE and ESIHE models is approximately equal to that of the proposed model. But these models suffer from noise and distortion. From Table 4, we can recognise that the proposed enhanced model is superior in enriching content, maintaining similarity, and suppressing the noise and distortion. The proposed enhanced image enhancement model generated a crisp and clear output.

### 3.2. Segmentation Comparison

**Figure 6:**
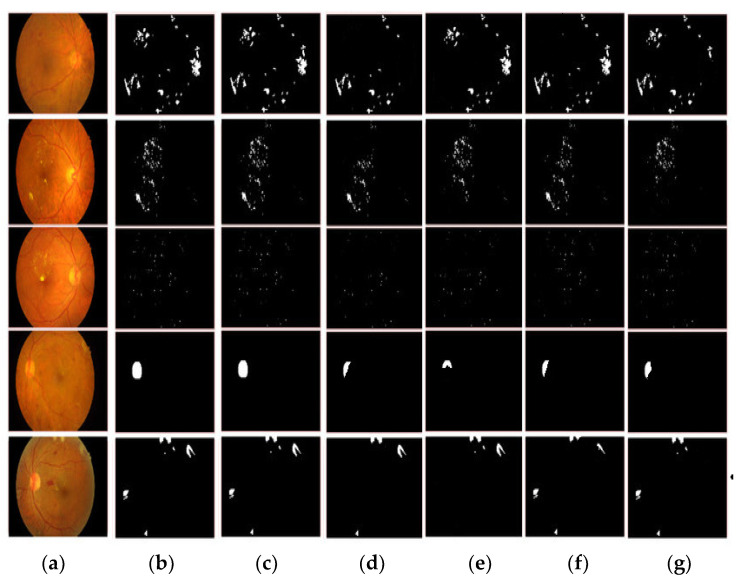
Segmentation results. (**a**) original image, (**b**) ground truth, (**c**) proposed model, (**d**) DenseNet, (**e**) Inception, (**f**) VGG-19, (**g**) AlexNet.

The proposed model obtains more accurate and robust segmentation results. From Figure 6 it can be noticed that the proposed model obtains more accurate results.

Table 5 tabulates the performance of the proposed enhanced ResNet-50 compared to the state-of-the-art models. The proposed system performed very accurately compared with the other lesion segmentation methods in the state-of-the-art models. It saves the obtained accuracy of abnormal fundus images. It achieves accurate, detailed segmentation results with small lesions, so it is the perfect choice for automatic computer-aided diagnosis (CAD) systems that depend on lesion segmentation results as it exceeds the estimations of the alternative models in terms of overall accuracy.

### 3.3. Evaluation of the APTOS Dataset

**Figure 7:**
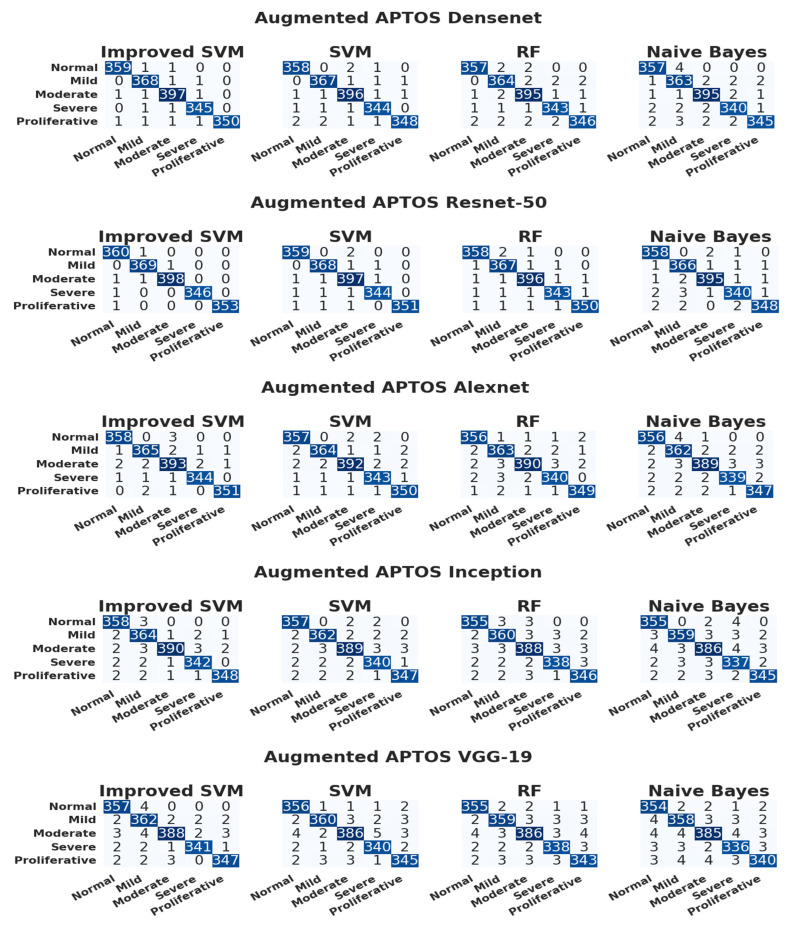
Confusion matrix for APTOS augmented dataset on different CNN models.

Figure 7 illustrates the confusion matrix for the APTOS dataset. We implemented five baseline models—VGG-16, DenseNet, ResNet-50, Xception, and AlexNet—and compared their performances on the APTOS dataset. From these five models, ResNet-50 showed the highest performance.

According to the 5-class confusion matrix mentioned above, the performance of each model was evaluated based on accuracy, recall, precision, and F1-score. Table 6 tabulates the APTOS fundus classification test set results. The improved SVM model achieved the highest accuracy of the remaining classification models. The results show that the augmented APTOS fundus classification for the ResNet-50 model achieves the highest accuracy for the improved SVM model.

### 3.4. Evaluation of the Kaggle Dataset

**Figure 8:**
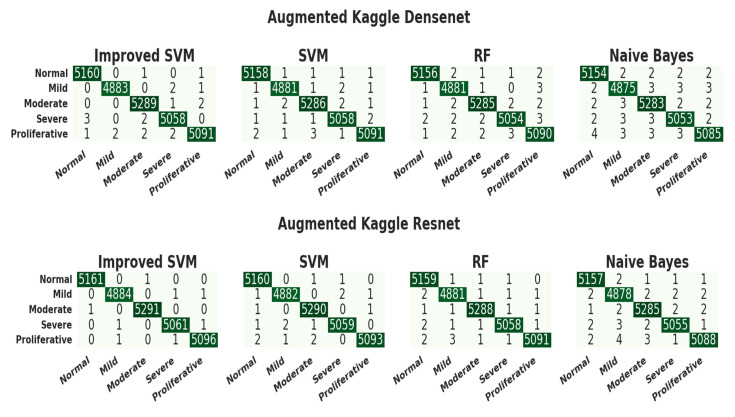

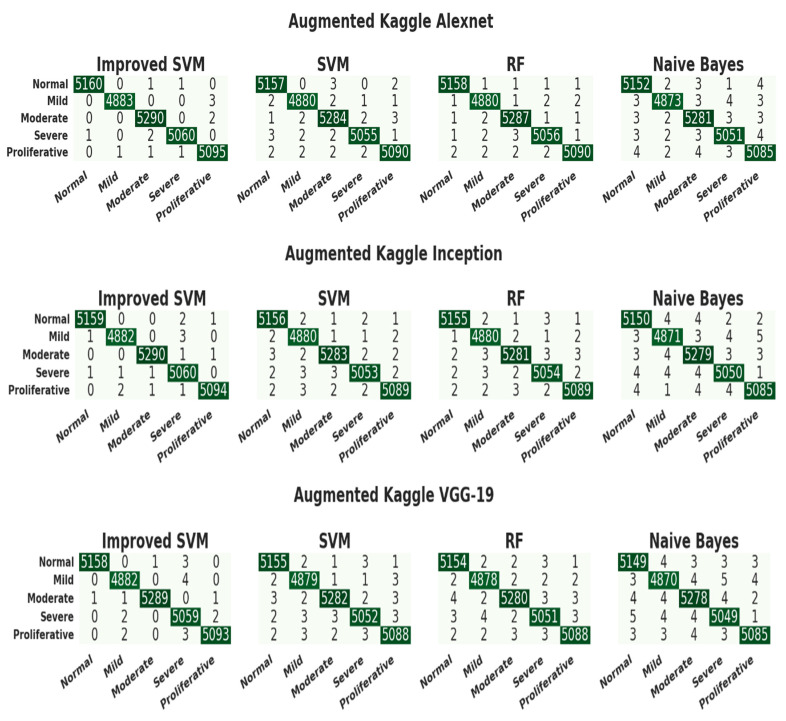
Confusion matrix for Kaggle augmented dataset on different CNN models.

Figure 8 illustrates the confusion matrix for the Kaggle dataset. We implemented five baseline models-VGG-16, DenseNet, ResNet-50, Xception, and AlexNet-and compared their performances on the Kaggle dataset. From these five models, ResNet-50 showed the highest performance. In 203 NODR fundus images, the proposed ISVM classifier accurately classified 202 fundus images for the ResNet-50 model. In 54 Mild images, the ISVM classifier accurately classified 54. Out of 69 moderate fundus images, ISVM accurately identified 68. Out of 15 images, ISVM accurately identified 14 for severe, and out of 7 images, ISVM accurately identified 6 for PDR for the ResNet-152 model. For the ResNet-50 model, the SVM classifier accurately identified 201 NODR images, 53 mild and 67 moderate, 14 severe, and 5 for PDR. For the ResNet-152 model, the RF classifier accurately identified 201 NODR images, 53 mild and 66 moderate, 13 severe, and 5 for PDR. For the ResNet-50 model, the NB classifier accurately identified 201 NODR images, 52 mild and 65 for moderate, 12 for severe, and 5 for PDR. Table 6 tabulated the Kaggle classification test set results.

From Table 7, we can see that the improved SVM model achieved the highest accuracy compared to the remaining classification models. The achieved results revealed that the overall testing accuracy and the performance metrics for the improved ResNet-50 with the improved SVM are the most appropriate for diabetic retinopathy detection, with a testing accuracy of 99.9% for fundus images.

**Figure 9:**
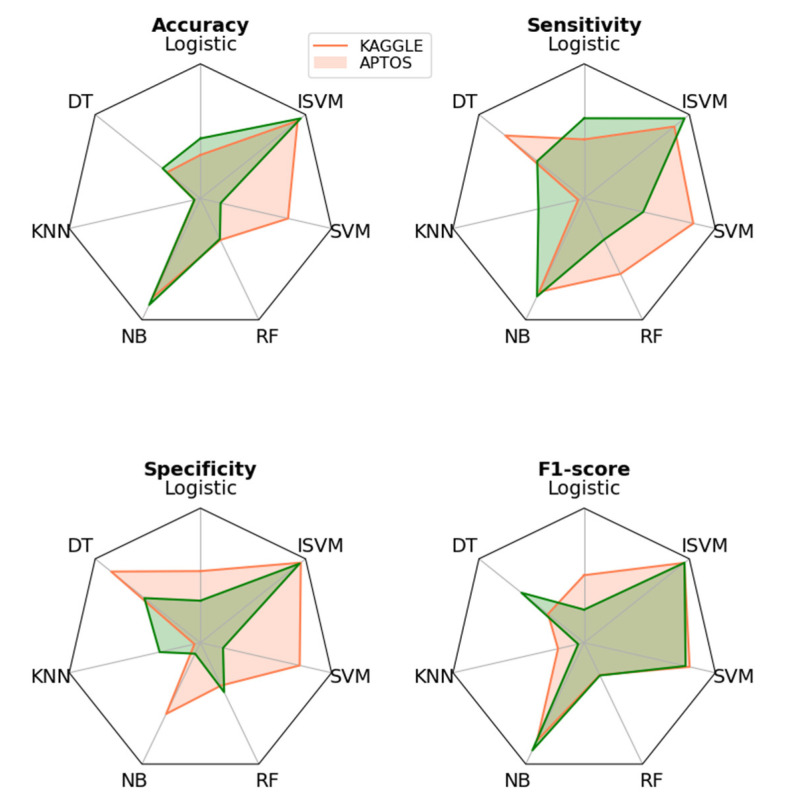
DR classification comparison of various classifiers of different datasets. It displays the performance results of the two datasets. Pink color represents the messidor and the green represents the APTOS.

Figure 9 presents the evaluation of the performance metrics for the different models. According to the achieved results, overall testing accuracy, and performance metrics, the proposed model is appropriate for detecting and classifying DR with a testing accuracy of 98.32% on the APTOS dataset.

Table 8 tabulates the varying sizes of the training and testing sets and the corresponding mean and standard deviation.

## 4. Discussion

This study aimed to identify and classify DR based on fundus images from two different datasets. Initially, all the images in the dataset were of different sizes; the images were resized to 225 × 225 using the RGB colour. The hyperparameters were tuned to optimize the proposed model. Model training can be accelerated, and the possibility of performance improved using the pooling function. There is no ideal batch size, and we implemented the experiments with various batch sizes. If we find the suitable batch size in addition to the suitable kernel and hidden layers, the model will yield a high performance. Batch size 64 produces better results than batch sizes 16 or 32. The batch size was 64 for the fundus images because this study’s dataset was large. From previous studies, we observed that the batch sizes, in conjunction with a suitable kernel and hidden layer, will yield a high performance. The parameters (i.e., a batch size of 64, epochs of 1000, and a learning rate of 0.001) were adjusted to achieve a high performance.

After extracting the features, the improved SVM classifies the lesions. In [15], the authors implemented AdaBoost to extract the features and the Gaussian mixture model, KNN, and SVM to classify the lesions and analyse the retina fundus images with different illuminations and views. A new unsupervised approach based on PCA for detecting microaneurysms was presented in [16]. The manual identification and differentiation of diabetic retinopathy from fundus images is time-consuming. Table 9 presents the processing time analysis of the existing techniques for the Kaggle and APTOS datasets to calculate the computation overhead. The achieved results revealed that the overall processing time for the improved SVM classifier is the most appropriate for diabetic retinopathy classification, with a minimum of 14 ms for Kaggle and 15 ms for APTOS datasets.

A study based on feature extraction using the RF model produced 74% accuracy in DR image classification [38]. Another two proposed hybrid models are based on combining the Gaussian mixture model and SVM to diagnose microaneurysms [18] and using KNN for the detection and classification of DR [39]. All the above-discussed studies used the existing classifiers to classify the DR lesions.

Some studies implemented CNN models to perform the binary classification of DR datasets [40,41]. Dropout regularization, augmentation, and pre-processing were performed manually by using the image editing tools in [42]. A deep CNN was proposed by [43] to classify normal and NPDR with two neural networks (i.e., the global and the local) and model performance was evaluated by the kappa score. The main disadvantage of this work is that it classifies only normal and NPPR, but it only works to detect the PDR.

To overcome those issues, the diagnostic results of the proposed model proved that it can achieve a satisfactory diagnostic performance, which can significantly assist the medical professional in the decision-making process in the early stages of detecting the infection, and timely treatment can decrease risk. Automatic screening and differentiation of diabetic retinopathy from fundus images will significantly reduce the effort of the medical professional and accelerate the diagnosis process.

Five class classifications are realized in the model, providing feasibility for the diagnosis of DR and its severity levels. The proposed model for the feature extraction and classification of DR performs better than the state-of-the-art models with high accuracy and less complexity. We will further optimize the model to model the accuracy of DR diagnosis and try to develop a more powerful DR detection model to assist doctors in clinical examinations.

The limitation of this model is that it is trained with only fundus image-level supervision, making it very challenging to accurately locate some minute lesion regions. Next, we need to specify the coarse location of the lesion along with the DR grading, which will help from the perspective of clinical application.

## 5. Conclusions

High blood pressure leads to DR, which causes retinal damage. Retinal vascularization is damaged by DR and can lead to blindness and potentially death. Fundoscopy examinations, which are time-consuming and expensive, allow ophthalmologists to see retinal vascular swelling. There is a need to automatically identify diabetic retinopathy by examining retinal fundus images. This study proposed an enhanced pooling function technique to minimize the loss to detect retina lesions, and an improved SVM classifier to classify the lesions using linear mapping. Five pre-trained deep learning models were recognized during the selection of the implementation, namely VGG-16, DenseNet, ResNet-50, Inception, and AlexNet. The proposed pooling and activation function results outperformed all the existing models. This study’s proposed model provided efficient accuracy results compared to the existing models.

## Figures and Tables

**Table 1:** Dataset distribution.

Dataset	NODR	Mild DR	Moderate DR	Severe DR	PDR	Count
APTOS	1805	370	999	193	295	3662
Kaggle	25,810	2443	5292	873	708	35,126

**Table 2:** Dataset augmentation operations.

Class	APTOS			Kaggle		
	Original	Operations	Augmented	Original	Operations	Augmented
NoDR	1805	0	1805	25,810	0	25,810
MildDR	370	5	1850	2443	10	24,430
Moderate DR	999	2	1998	5292	5	26,460
Severe DR	193	9	1737	873	29	25,317
PDR	295	6	1770	708	36	25,488
Total	3662		9160	35,126		127,505

**Table 3:** Augmented dataset image distribution.

Class	APTOS		Kaggle	
	Training	Testing	Training	Testing
NoDR	1444	361	20,648	5162
MildDR	1480	370	19,544	4886
Moderate DR	1598	400	21,166	5292
Severe DR	1390	347	20,254	5063
PDR	1416	354	20,390	5098
Total	7328	1832	102,004	25,501

**Table 4:** Average scores for the augmented APTOS dataset.

Model	PSNR	GMSD	Entropy	SSIM	PCQI	Processing Time (s)
Clahe [24]	30.83	0.163	7.263	0.634	1.139	0.155
ESIHE [25]	31.93	0.074	7.316	0.635	1.282	0.153
HAHE [26]	32.82	0.125	7.226	0.693	1.001	0.373
BIMEF [27]	31.68	0.199	7.269	0.736	1.007	0.364
HMF [28]	32.63	0.085	7.283	0.636	1.103	0.218
ABC	34.83	0.048	7.834	0.877	1.378	0.173
Proposed	35.56	0.037	7.935	0.983	1.484	0.151

**Table 5:** Comparison of segmentation results for the APTOS dataset with the state-of-the-art models.

Model	Pool + Act	Accuracy	Precision	Recall
DenseNet [29]	Max + Relu	0.9484	0.8364	0.9584
Inception [12]	Max + Relu	0.9847	0.8578	0.9848
VGG-19 [30]	Max + Relu	0.9795	0.8479	0.9483
AlexNet [31]	Max + Relu	0.9858	0.9378	0.9847
ResNet-50	Proposed	0.9986	1.0000	1.0000
AlexNet	Proposed	0.9986	1.0000	0.9864
DenseNet	Proposed	0.9959	1.0000	0.9916
Inception	Proposed	0.9972	0.9864	0.9864
VGG-19	Proposed	0.9986	0.9866	1.0000

**Table 6:** Performance metrics for APTOS augmented dataset.

CNN Model	Classifier	Accuracy	Precision	Recall	F1-Score	Class
DenseNet	ISVM	0.99781659	0.99445983	0.99445983	0.99445983	Normal
		0.99672489	0.98924731	0.99459459	0.99191375	Mild
		0.99617904	0.99002494	0.99250000	0.99126092	Moderate
		0.99727074	0.99137931	0.99423631	0.99280576	Severe
		0.99781659	1.0000000	0.98870056	0.99431818	PDR
	SVM	0.99617904	0.98895028	0.99168975	0.99031812	Normal
		0.99617904	0.98921833	0.99189189	0.99055331	Mild
		0.99508734	0.98753117	0.99000000	0.98876404	Moderate
		0.99617904	0.98850575	0.99135447	0.98992806	Severe
		0.99563319	0.99428571	0.98305085	0.98863636	PDR
	RF	0.99563319	0.98891967	0.98891967	0.98891967	Normal
		0.99290393	0.98113208	0.98378378	0.98245614	Mild
		0.99344978	0.98258706	0.98750000	0.98503741	Moderate
		0.99508734	0.98563218	0.98847262	0.98705036	Severe
		0.99344978	0.98857143	0.97740113	0.98295455	PDR
	NB	0.99454148	0.98347107	0.98891967	0.98618785	Normal
		0.99072052	0.97319035	0.98108108	0.97711978	Mild
		0.99399563	0.98503741	0.98750000	0.98626717	Moderate
		0.99290393	0.98265896	0.97982709	0.98124098	Severe
		0.99290393	0.98853868	0.97457627	0.98150782	PDR
ResNet-50	ISVM	0.99781659	0.99173554	0.99722992	0.99447514	Normal
		0.99836245	0.99460916	0.9972973	0.99595142	Mild
		0.99836245	0.99749373	0.9950000	0.99624531	Moderate
		0.99945415	1.00000000	0.99711816	0.99855700	Severe
		0.99945415	1.00000000	0.99717514	0.99858557	PDR
	SVM	0.99727074	0.99171271	0.99445983	0.99308437	Normal
		0.99727074	0.99191375	0.99459459	0.99325236	Mild
		0.99563319	0.98756219	0.99250000	0.99002494	Moderate
		0.99727074	0.99421965	0.99135447	0.99278499	Severe
		0.99836245	1.00000000	0.99152542	0.99574468	PDR
	RF	0.99617904	0.98895028	0.99168975	0.99031812	Normal
		0.99563319	0.98655914	0.99189189	0.98921833	Mild
		0.99563319	0.99000000	0.99000000	0.99000000	Moderate
		0.99617904	0.99132948	0.98847262	0.98989899	Severe
		0.99672489	0.99431818	0.98870056	0.99150142	PDR
	NB	0.99508734	0.98351648	0.99168975	0.98758621	Normal
		0.99399563	0.98123324	0.98918919	0.98519515	Mild
		0.99508734	0.98997494	0.98750000	0.98873592	Moderate
		0.99344978	0.98550725	0.97982709	0.98265896	Severe
		0.99508734	0.99145299	0.98305085	0.98723404	PDR
AlexNet	ISVM	0.99617904	0.98895028	0.99168975	0.99031812	Normal
		0.99454148	0.98648649	0.98648649	0.98648649	Mild
		0.99235808	0.98250000	0.98250000	0.98250000	Moderate
		0.99672489	0.99135447	0.99135447	0.99135447	Severe
		0.99727074	0.99433428	0.99152542	0.99292786	PDR
	SVM	0.99454148	0.98347107	0.98891967	0.98618785	Normal
		0.99454148	0.98913043	0.98378378	0.98644986	Mild
		0.99290393	0.98740554	0.98000000	0.98368883	Moderate
		0.99454148	0.98280802	0.98847262	0.98563218	Severe
		0.99508734	0.98591549	0.98870056	0.98730606	PDR
	RF	0.99344978	0.98071625	0.98614958	0.98342541	Normal
		0.99126638	0.97580645	0.98108108	0.97843666	Mild
		0.99126638	0.98484848	0.97500000	0.97989950	Moderate
		0.99235808	0.97982709	0.97982709	0.97982709	Severe
		0.99454148	0.98587571	0.98587571	0.98587571	PDR
	NB	0.99290393	0.97802198	0.98614958	0.98206897	Normal
		0.98962882	0.97050938	0.97837838	0.97442799	Mild
		0.99017467	0.98232323	0.97250000	0.97738693	Moderate
		0.99235808	0.9826087	0.97694524	0.97976879	Severe
		0.99235808	0.98022599	0.98022599	0.98022599	PDR
Inception	ISVM	0.99399563	0.97814208	0.99168975	0.98486933	Normal
		0.99126638	0.97326203	0.98378378	0.97849462	Mild
		0.99290393	0.99236641	0.97500000	0.98360656	Moderate
		0.99399563	0.98275862	0.98559078	0.98417266	Severe
		0.99508734	0.99145299	0.98305085	0.98723404	PDR
	SVM	0.99344978	0.97808219	0.98891967	0.98347107	Normal
		0.99181223	0.98102981	0.97837838	0.97970230	Mild
		0.98962882	0.97984887	0.97250000	0.97616060	Moderate
		0.99181223	0.97701149	0.97982709	0.97841727	Severe
		0.99290393	0.98300283	0.98022599	0.98161245	PDR
	RF	0.99181223	0.97527473	0.98337950	0.97931034	Normal
		0.98908297	0.97297297	0.97297297	0.97297297	Mild
		0.98744541	0.97243108	0.9700000	0.97121402	Moderate
		0.99126638	0.97971014	0.9740634	0.97687861	Severe
		0.99126638	0.97740113	0.97740113	0.97740113	PDR
	NB	0.99072052	0.96994536	0.98337950	0.97661623	Normal
		0.98962882	0.97820163	0.97027027	0.97421981	Mild
		0.98635371	0.97229219	0.96500000	0.96863237	Moderate
		0.98744541	0.96285714	0.97118156	0.96700143	Severe
		0.99126638	0.98011364	0.97457627	0.97733711	PDR
VGG-19	ISVM	0.99290393	0.97540984	0.98891967	0.98211829	Normal
		0.98908297	0.96791444	0.97837838	0.97311828	Mild
		0.99017467	0.98477157	0.97000000	0.97732997	Moderate
		0.99454148	0.98840580	0.98270893	0.98554913	Severe
		0.99290393	0.98300283	0.98022599	0.98161245	PDR
	SVM	0.99181223	0.97267760	0.98614958	0.97936726	Normal
		0.99072052	0.98092643	0.97297297	0.97693351	Mild
		0.98744541	0.97721519	0.96500000	0.97106918	Moderate
		0.99126638	0.97421203	0.97982709	0.97701149	Severe
		0.98962882	0.97183099	0.97457627	0.97320169	PDR
	RF	0.99126638	0.97260274	0.98337950	0.97796143	Normal
		0.98853712	0.97289973	0.97027027	0.97158322	Mild
		0.98689956	0.97474747	0.96500000	0.96984925	Moderate
		0.98962882	0.97126437	0.97406340	0.97266187	Severe
		0.98799127	0.96892655	0.96892655	0.96892655	PDR
	NB	0.98853712	0.96195652	0.98060942	0.97119342	Normal
		0.98635371	0.96495957	0.96756757	0.96626181	Mild
		0.98580786	0.97222222	0.96250000	0.96733668	Moderate
		0.98799127	0.96829971	0.96829971	0.96829971	Severe
		0.98689956	0.97142857	0.96045198	0.96590909	PDR

**Table 7:** Performance metrics for Kaggle augmented dataset.

CNN Model	Classifier	Accuracy	Precision	Recall	F1-Score	Class
DenseNet	ISVM	0.99976472	0.99922541	0.99961255	0.99941894	Normal
		0.99980393	0.99959058	0.99938600	0.99948828	Mild
		0.99968629	0.99905553	0.99943311	0.99924428	Moderate
		0.99960786	0.99901244	0.99901244	0.99901244	Severe
		0.99956864	0.99921492	0.99862691	0.99892083	PDR
	SVM	0.99964707	0.99903157	0.99922511	0.99912833	Normal
		0.99960786	0.99897667	0.99897667	0.99897667	Mild
		0.99952943	0.99886621	0.99886621	0.99886621	Moderate
		0.99956864	0.99881517	0.99901244	0.99891379	Severe
		0.99952943	0.99901884	0.99862691	0.99882284	PDR
	RF	0.99956864	0.99903120	0.99883766	0.99893442	Normal
		0.99949022	0.99836367	0.99897667	0.99867008	Mild
		0.99949022	0.99886600	0.99867725	0.99877161	Moderate
		0.99941179	0.99881423	0.9982224	0.99851823	Severe
		0.99929415	0.99803922	0.99843076	0.99823495	PDR
	NB	0.99929415	0.99806352	0.99845021	0.99825683	Normal
		0.99913729	0.99774867	0.99774867	0.99774867	Mild
		0.99921572	0.99792218	0.99829932	0.99811071	Moderate
		0.99921572	0.99802489	0.99802489	0.99802489	Severe
		0.99913729	0.99823322	0.99744998	0.99784144	PDR
ResNet-50	ISVM	0.99992157	0.99980628	0.99980628	0.99980628	Normal
		0.99984314	0.99959067	0.99959067	0.99959067	Mild
		0.99992157	0.99981104	0.99981104	0.99981104	Moderate
		0.99984314	0.99960498	0.99960498	0.99960498	Severe
		0.99984314	0.99960769	0.99960769	0.99960769	PDR
	SVM	0.99972550	0.99903195	0.99961255	0.99932217	Normal
		0.99972550	0.99938588	0.99918133	0.99928359	Mild
		0.99976472	0.99924443	0.99962207	0.99943321	Moderate
		0.99972550	0.99940735	0.99920995	0.99930864	Severe
		0.99972550	0.99960746	0.99901922	0.99931325	PDR
	RF	0.99960786	0.99864499	0.99941883	0.99903176	Normal
		0.99956864	0.99877225	0.99897667	0.99887445	Mild
		0.99968629	0.99924414	0.99924414	0.99924414	Moderate
		0.99964707	0.99920980	0.99901244	0.99911111	Severe
		0.99960786	0.99941107	0.99862691	0.99901884	PDR
	NB	0.99952943	0.99864446	0.99903138	0.99883788	Normal
		0.99925493	0.99775005	0.99836267	0.99805627	Mild
		0.99941179	0.99848857	0.99867725	0.99858290	Moderate
		0.99945100	0.99881446	0.99841991	0.99861715	Severe
		0.99937257	0.99882214	0.99803845	0.99843014	PDR
AlexNet	ISVM	0.99988236	0.99980624	0.99961255	0.99970939	Normal
		0.99984314	0.99979525	0.99938600	0.99959058	Mild
		0.99976472	0.99924443	0.99962207	0.99943321	Moderate
		0.99980393	0.99960490	0.99940747	0.99950617	Severe
		0.99968629	0.99901961	0.99941153	0.99921553	PDR
	SVM	0.99949022	0.99845111	0.99903138	0.99874116	Normal
		0.99952943	0.99877200	0.99877200	0.99877200	Mild
		0.99933336	0.99829964	0.99848828	0.99839395	Moderate
		0.99949022	0.99901186	0.99841991	0.99871580	Severe
		0.99941179	0.99862664	0.99843076	0.99852869	PDR
	RF	0.99964707	0.99903157	0.99922511	0.99912833	Normal
		0.99949022	0.99856763	0.99877200	0.99866980	Mild
		0.99952943	0.99867775	0.99905518	0.99886643	Moderate
		0.99949022	0.99881470	0.99861742	0.99871605	Severe
		0.99949022	0.99901865	0.99843076	0.99872461	PDR
	NB	0.99909807	0.99748306	0.99806277	0.99777283	Normal
		0.99917650	0.99836099	0.99733934	0.99784990	Mild
		0.99905886	0.99754439	0.99792139	0.99773285	Moderate
		0.99909807	0.99782695	0.99762986	0.99772840	Severe
		0.99894122	0.99725436	0.99744998	0.99735216	PDR
Inception	ISVM	0.99980393	0.99961248	0.99941883	0.99951564	Normal
		0.99972550	0.99938588	0.99918133	0.99928359	Mild
		0.99984314	0.99962207	0.99962207	0.99962207	Moderate
		0.99960786	0.99861851	0.99940747	0.99901283	Severe
		0.99976472	0.99960754	0.99921538	0.99941142	PDR
	SVM	0.99941179	0.99825750	0.99883766	0.99854750	Normal
		0.99937257	0.99795501	0.99877200	0.99836334	Mild
		0.99937257	0.99867675	0.99829932	0.99848800	Moderate
		0.99933336	0.99861660	0.99802489	0.99832066	Severe
		0.99937257	0.99862637	0.99823460	0.99843045	PDR
	RF	0.99945100	0.99864394	0.99864394	0.99864394	Normal
		0.99937257	0.99795501	0.99877200	0.99836334	Mild
		0.99925493	0.99848743	0.99792139	0.99820433	Moderate
		0.99929415	0.99822240	0.9982224	0.99822240	Severe
		0.99933336	0.99843045	0.9982346	0.99833252	PDR
	NB	0.99898043	0.99728892	0.99767532	0.99748208	Normal
		0.99890200	0.99733825	0.99693000	0.99713408	Mild
		0.99890200	0.99716660	0.99754346	0.99735500	Moderate
		0.99898043	0.99743235	0.99743235	0.99743235	Severe
		0.99905886	0.99784144	0.99744998	0.99764567	PDR
VGG-19	ISVM	0.99980393	0.99980616	0.99922511	0.99951555	Normal
		0.99964707	0.99897688	0.99918133	0.99907910	Mild
		0.99984314	0.99981096	0.99943311	0.99962200	Moderate
		0.99945100	0.99802722	0.99920995	0.99861824	Severe
		0.99968629	0.99941130	0.99901922	0.99921522	PDR
	SVM	0.99937257	0.99825716	0.99864394	0.99845051	Normal
		0.99933336	0.99795459	0.99856734	0.99826087	Mild
		0.99933336	0.99867650	0.99811036	0.99839335	Moderate
		0.99921572	0.99822170	0.99782738	0.99802450	Severe
		0.99921572	0.99803845	0.99803845	0.99803845	PDR
	RF	0.99925493	0.99787028	0.99845021	0.99816016	Normal
		0.99929415	0.99795417	0.99836267	0.99815838	Mild
		0.99917650	0.99829836	0.99773243	0.99801531	Moderate
		0.99909807	0.99782695	0.99762986	0.99772840	Severe
		0.99925493	0.99823426	0.99803845	0.99813634	PDR
	NB	0.99890200	0.99709527	0.99748160	0.99728840	Normal
		0.99878436	0.99692938	0.99672534	0.99682735	Mild
		0.99886279	0.99716607	0.99735450	0.99726027	Moderate
		0.99886279	0.99703791	0.99723484	0.99713637	Severe
		0.99909807	0.99803729	0.99744998	0.99774355	PDR

**Table 8:** Varying training and test size.

Dataset	Training	Testing	Accuracy	Mean	Standard Deviation
APTOS	70	30	0.981225	0.982543	0.0011409
	75	25	0.983202		
	80	20	0.983202		
Kaggle	70	30	0.971344	0.980237	0.0080882
	75	25	0.982213		
	80	20	0.987154		

**Table 9:** DR classification comparison of the processing time for the proposed model with different optimizations.

Classifier	Kaggle (s)	APTOS (s)
Logistic regression [32]	21	29
DT [33]	15	21
KNN [34]	23	30
NB [35]	20	25
RF [36]	20	23
SVM [37]	22	31
Improved SVM	14	15

## Data Availability

Not applicable.
